# Oxygen consumption measurements at ultra‐high dose rate over a wide LET range

**DOI:** 10.1002/mp.17496

**Published:** 2024-11-06

**Authors:** Celine Karle, Hans Liew, Thomas Tessonnier, Stewart Mein, Kristoffer Petersson, Christian Schömers, Stefan Scheloske, Stephan Brons, Rainer Cee, Gerald Major, Thomas Haberer, Amir Abdollahi, Jürgen Debus, Ivana Dokic, Andrea Mairani

**Affiliations:** ^1^ Clinical Cooperation Unit Translational Radiation Oncology National Center for Tumor Diseases (NCT) Heidelberg University Hospital (UKHD) and German Cancer Research Center (DKFZ) Heidelberg Germany; ^2^ Department of Physics and Astronomy Heidelberg University Heidelberg Germany; ^3^ Division of Molecular and Translational Radiation Oncology, Heidelberg Faculty of Medicine (MFHD) and Heidelberg University Hospital (UKHD) Heidelberg Ion‐Beam Therapy Center (HIT) Heidelberg Germany; ^4^ Heidelberg Institute of Radiation Oncology (HIRO) National Center for Radiation Oncology (NCRO) Heidelberg University and German Cancer Research Center (DKFZ) Heidelberg Germany; ^5^ Heidelberg Ion‐Beam Therapy Center (HIT), Department of Radiation Oncology Heidelberg University Hospital Heidelberg Germany; ^6^ Clinical Cooperation Unit Radiation Oncology, National Center for Tumor Diseases (NCT) Heidelberg University Hospital (UKHD) and German Cancer Research Center (DKFZ) Heidelberg Germany; ^7^ Department of Radiation Oncology University of Pennsylvania Philadelphia Pennsylvania USA; ^8^ Oxford Institute for Radiation Oncology Department of Oncology Old Road Campus Research Building University of Oxford Oxford UK; ^9^ Department of Radiation Oncology, Heidelberg University Hospital, Heidelberg Institute of Radiation Oncology (HIRO), University Hospital Heidelberg, National Center for Tumor Diseases (NCT) University Hospital Heidelberg Heidelberg Germany; ^10^ National Center for Oncological Hadrontherapy (CNAO) Medical Physics Pavia Italy

**Keywords:** heavy ions, LET, oxygen consumption

## Abstract

**Background:**

The role of radiolytic oxygen consumption for the in‐vitro “Ultra‐High Dose Rate” (UHDR) sparing and in‐vivo FLASH effect is subject to active debate, but data on key dependencies such as the radiation quality are lacking.

**Purpose:**

The influence of “dose‐averaged Linear Energy Transfer” (LETd) and dose rate on radiolytic oxygen consumption was investigated by monitoring the oxygen concentration during irradiation with electrons, protons, helium, carbon, and oxygen ions at UHDR and “Standard Dose Rates” (SDR).

**Methods:**

Sealed “Bovine Serum Albumin” (BSA) 5% samples were exposed to 15 Gy of electrons and protons, and for the first time helium, carbon, and oxygen ions with LETd values of 1, 5.4, 14.4, 65, and 100.3 keV/µm, respectively, delivered at mean dose rates of either 0.3–0.4 Gy/s for SDR or approximately 100 Gy/s for UHDR. The Oxylite (Oxford Optronics) system allowed measurements of the oxygen concentration before and after irradiation to calculate the oxygen consumption rate.

**Results:**

The oxygen consumption rate was found to decrease with increasing LETd from 0.351 mmHg/Gy for low LET electrons to 0.1796 mmHg/Gy for high LET oxygen ions at SDR and for UHDR from 0.317 to 0.1556 mmHg/Gy, respectively. A higher consumption rate for SDR irradiation compared to the corresponding UHDR irradiation persisted for all particle types.

**Conclusion:**

The measured consumption rates demonstrate a distinct LETd dependence. The obtained dataset, encompassing a wide range of LETd values, could serve as a benchmark for Monte Carlo simulations, which may aid in enhancing our comprehension of oxygen‐related mechanisms after irradiations. Ultimately, they could help assess the viability of different hypotheses regarding UHDR sparing mechanisms and the FLASH effect. The found LETd dependence underscores the potential of heavy ion therapy, wherein elevated consumption rates in adjacent normal tissue offer protective benefits, while leaving tumor regions with generally higher “Linear Energy Transfer” (LET) vulnerable.

## 1 INTRODUCTION

Since the 1920s, the pivotal role of oxygen concentration in influencing cellular sensitivity to radiation and, consequently, radiotherapy outcomes has been recognized.^1^ When incident radiation interacts with the water molecules, free radicals are produced, which are important contributors to the induction of DNA damage. Subsequent chemical reactions consume the present molecular oxygen.^2^, ^3^ The radiolytic reaction cascades and oxygen concentration are linked to the physical beam parameters, specifically, dose rate, and “dose‐averaged Linear Energy Transfer” (LETd).^4^, ^5^, ^6^

The recent resurgence of interest in the oxygen effect emerged mainly by the first reports of the so‐called FLASH effect in 2014.^7^ The FLASH effect describes the differential response of normal and tumor tissue in vivo to “Ultra‐High Dose Rate” (UHDR) irradiation above 40 Gy/s, with the former being spared and the latter being treated iso‐effectively compared to the same dose irradiated at “Standard Dose Rates” (SDR) approximately 0.001–0.5 Gy/s.^8^ However, the underlying mechanisms of the in vivo FLASH effect remain to be elucidated.

As early as 1959, in vitro experiments to investigate dose‐rate‐dependent mechanisms showed an increase in cell survival rates after UHDR irradiation.^6^ On this cellular level, the extent of UHDR sparing was generally found to be affected by the oxygen concentration within the system leading many to believe that oxygen and the oxygen consumption processes play a key role in the underlying sparing mechanism.^9^, ^10^, ^11^, ^12^

The “oxygen depletion hypothesis” postulates that the reactions induced by UHDR radiation could consume oxygen, resulting in a transient state of oxygen deprivation that could protect the cells.^9^, ^11^ Since it takes about 50 ms to unload oxygen from oxyhemoglobin alone, re‐oxygenation begins long after the free radical chemistry has started after the incident UHDR irradiation, and the cells remain in a state of oxygen depletion.^13^ Consequently, during prolonged SDR irradiation where the irradiation timescale could be in the same range or even longer than the reoxygenation timescale, reoxygenation could counteract the oxygen consumption during the irradiation time.^12^ This explanation has been criticized since the radiation‐induced decrease in oxygen concentration may not be sufficient to induce the hypoxic state that would explain the overall sparing effect.^14^, ^15^

Another contributing factor might be altered radical‐radical interactions. After UHDR exposure, the reactions occur in closer spatiotemporal proximity, which promotes radical‐radical interactions.

A common feature of the proposed mechanisms is that the dynamics of the chemical reactions differ between UHDR and SDR irradiation.^16^ Therefore, direct measurements of the outcome of these reactions, like the changes in oxygen concentration, are crucial for assessing the plausibility of these approaches and understanding irradiation‐induced reactions with oxygen. Such measurements have already been conducted in deionized water^15^, ^17^ and solutions closer to biological systems,^17^ such as “Bovine Serum Albumin” (BSA)^14^ or CELL,^18^ a solution mimicking the intracellular environment. The studies conducted in such surrogate media using electron or proton beams consistently found that the UHDR oxygen consumption rate is smaller than the corresponding SDR value. For UHDR irradiation with heavier ions, so far only in silico studies have explored the additional factor of LETd.^19^, ^20^

In this study, we conducted a comprehensive examination of oxygen consumption measurements at various LETds up to 100.3 keV/µm. Beyond electrons and protons, we, for the first time, irradiated BSA 5% samples additionally with helium, carbon, and oxygen ions at UHDR and SDR. We discuss the reasons behind observed oxygen consumption trends as well as the potential implications for FLASH dose rates in heavy ion therapy and their contribution to understanding the underlying mechanisms of UHDR sparing.

## 2 MATERIAL AND METHODS

### 2.1 Sample preparation

Prior to each irradiation, a fresh BSA 5% stock was prepared with BSA from ROTH (Art.‐Nr. 0163.3) dissolved in DPBS from Gibco (Kat.Nr. 14190250). The air‐equilibrated stock was filled into 2 mL Eppendorf tubes. The electron irradiation field covered the entire sample, while for irradiations at the “Heidelberg Ion‐Beam Therapy Center” (HIT), the Eppendorf tubes were cut off at 1 mL to fit into the 2 cm “Spread‐Out Bragg Peak” (SOBP). The Eppendorf tubes were sealed with parafilm while maintaining a bubble‐free environment to inhibit reoxygenation.

### 2.2 Oxygen measurement

The oxygen measurements during irradiation were conducted with the OxyLite system (Oxford Optronix). The NX‐BF/OT/E Oxygen/Temperature bare‐fiber sensor attached to the OxyLite system measures the lifetime of fluorescence in ruthenium luminophore, located in the silicon rubber polymer of the probe tip. Since the presence of oxygen quenches the fluorescence lifetime, the latter is inversely proportional to the oxygen concentration. Using this method, the oxygen concentration in the liquid is determined.^21^ OxyLite was shown to provide reliable oxygen concentration values^21^ with 10% accuracy in the range of 7– 150 mmHg, and below 7 mmHg an accuracy of ± 0.7 mmHg. All used sensors were pre‐calibrated and possess an internal temperature compensation.

### 2.3 Irradiation setup and dosimetry

**FIGURE 1:**
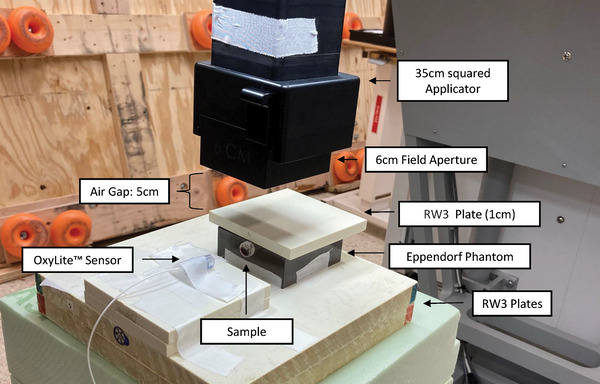
Experimental setup at the Mobetron for electron SDR and UHDR irradiation of the Eppendorf tubes.

For electron irradiation, the Mobetron from IntraOp was utilized (Figure 1). The dose rate of the 9 MeV beam was modified by adjusting the pulse repetition frequency and pulse length (Table 1). The dosimetry mimicking the required setup was conducted with the “FLASH‐µDiamond” (PTW, SN:7602), which was cross‐calibrated with an Advanced Marcus chamber (PTW, REF: TM34045, SN:0535).

**TABLE 1:** Dosimetry measurements and setting details for electron SDR and UHDR irradiation with the Mobetron (IntraOp) for the planned dose of 15 Gy. The dose values are averages of at least three values and standard deviations are given.

Field diameter (cm)	Source‐surface distance (cm)	Extraction energy (MeV)	Mean LETd (keV/µm)	Mean dose rate (Gy/s)	Pulse repetition frequency (Hz)	Puls length (µs)	Radiation unit	Dose (Gy)
6	40	9	1	0.338 ± 0.003	30	1.2	707 Monitor Units	15.011 ± 0.005
				112.2 ± 0.9	45	4	6 Pulses	15.01 ± 0.18

The electrons LETd was estimated to be 1 keV/µm.^22^

The samples were horizontally positioned within a dedicated phantom and uniformly irradiated from above by a circular field with a diameter of 6 cm (Figure 1).

**FIGURE 2:**
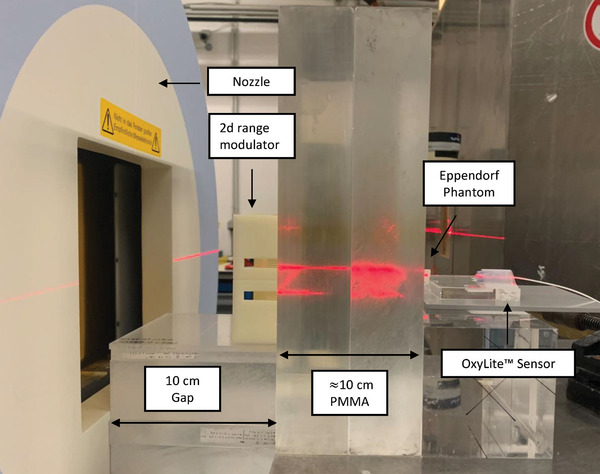
Experimental setup at the HIT beamline for SDR and UHDR irradiation of the Eppendorf tubes with protons, helium, carbon, and oxygen ions.

The active raster scanning delivery of protons (p), helium (^4^He), carbon (^12^C), and oxygen (^16^O) ions was performed at the HIT experimental room (Figure 2 and Table 2). The LETd of the p, ^4^He, ^12^C, and ^16^O beams were estimated using FLUKA “Monte Carlo” (MC) simulations^23^, ^24^ to be approx. 5.4 (range in the region of interest 4.9–6.0 keV/µm) keV/µm, 14.4 (12–33) keV/µm, 65 (56–153) keV/µm, and 100.3 (88–235) keV/µm, respectively. Daily dosimetric measurements were conducted with a PinPoint ionization chamber (PTW, REF: TM31015, SN:0903)^25^ following clinical practice TRS398 to guarantee a correct dose application. Additionally, Gafchromic EBT3 films (8″X10″ (x25), Ashland 828204) were irradiated to ensure the homogeneity of the fields. The monitoring chamber in the beam nozzle was flushed with 96%/4% helium/CO_2_ gas mixture, to prevent saturation during UHDR irradiations.^26^ Therewith, the spill length and its delivery could be monitored and evaluated (Table 2). All plans have a field size larger than 9 × 9 mm^2^ to ensure full coverage of the Eppendorf tubes. The spot spacings were adapted to the different lateral scattering properties of the particles. To ensure comparability between UHDR and SDR plans, the field size and spot spacing patterns were kept identical for each ion. Only two key parameters were changed between the two modes, namely the beam current and the number of spills. First, to bunch all particles into one single spill for the UHDR irradiation, the requested beam current was increased by a factor of approximately 100. This enables a faster particle extraction using a stronger radiofrequency‐knockout exciter amplitude.^27^ Second, the total amount of particles from the UHDR plan was split into 10 separate spills for the SDR. Therewith, a clinical SDR of about 0.3–0.4 Gy/s on average was achieved.^28^

**TABLE 2:** Field and irradiation parameter details for SDR and UHDR irradiation at the HIT facility.

Particle	Field size ($mm^{2}$)	Spot spacing (mm)	Mean LETd (keV/µm)	Extraction energy (MeV/u)	Mean dose rate (Gy/s)	Delivery time (s)	Spill time (ms)	Interspill time (s)	Dose (Gy)
p	12	3	5.4 [range 4.9–6.0]	146.56	0.40 ± 0.03	38 ± 3	533 ± 67	3.46 ± 0.03	14.997 ± 0.026
					121 ± 8	0.125 ± 0.008	125 ± 8	–	15.08 ± 0.03
^4^He	10	2	14.4 [range 12.0–33.0]	145.74	0.4074 ± 0.0015	36.82 ± 0.12	336.0 ± 1.7	3.654 ± 0.004	15.003 ± 0.015
					121 ± 6	0.125 ± 0.006	125 ± 6	–	15.07 ± 0.12
^12^C	10.5	1.5	65 [range 56–153]	275.98	0.341 ± 0.009	44.6 ± 1.2	696 ± 115	4.5 ± 0.8	15.21 ± 0.11
					113 ± 8	0.136 ± 0.009	136 ± 9	–	15.27 ± 0.12
^16^O	9	1.5	100.3 [range 88.0–235.0]	325.98	0.309 ± 0.010	48.5 ± 1.6	876 ± 154	4.39 ± 0.05	15.02 ± 0.04
					108 ± 10	0.138 ± 0.012	137 ± 12	–	14.86 ± 0.16

All values are averaged from at least three samples and are given with their standard deviations. The “Delivery Time” describes the time span from the beginning of the first spill to the end of the last one, while the “Interspill Time” lasts from the end of one spill until the beginning of the following spill. The “Spill Time” refers to the temporal length of a single spill. The “Mean Dose Rate” is calculated by dividing the delivered dose by the delivery time.

To employ the clinically established active raster beam scanning delivery technique, it is necessary to extend the pristine Bragg peak from the delivered monoenergetic beams to cover the entire sample during a single spill. For this purpose, a “2‐Dimensional Range Modulator” (2DRM) was used.^29^, ^30^ The 10 × 10 cm^2^ 2DRM contains 50 × 50 pins with a length of 20 mm to produce a 2 cm SOBP.^29^, ^30^ Depth dose curves were assessed for each particle (Figure S1).^28^ The samples were placed horizontally into a dedicated “Poly(methyl methacrylate)” (PMMA) phantom and slices of PMMA were positioned in front of the samples (Figure 2).^28^

### 2.4 Data evaluation and statistical analysis

**FIGURE 3:**
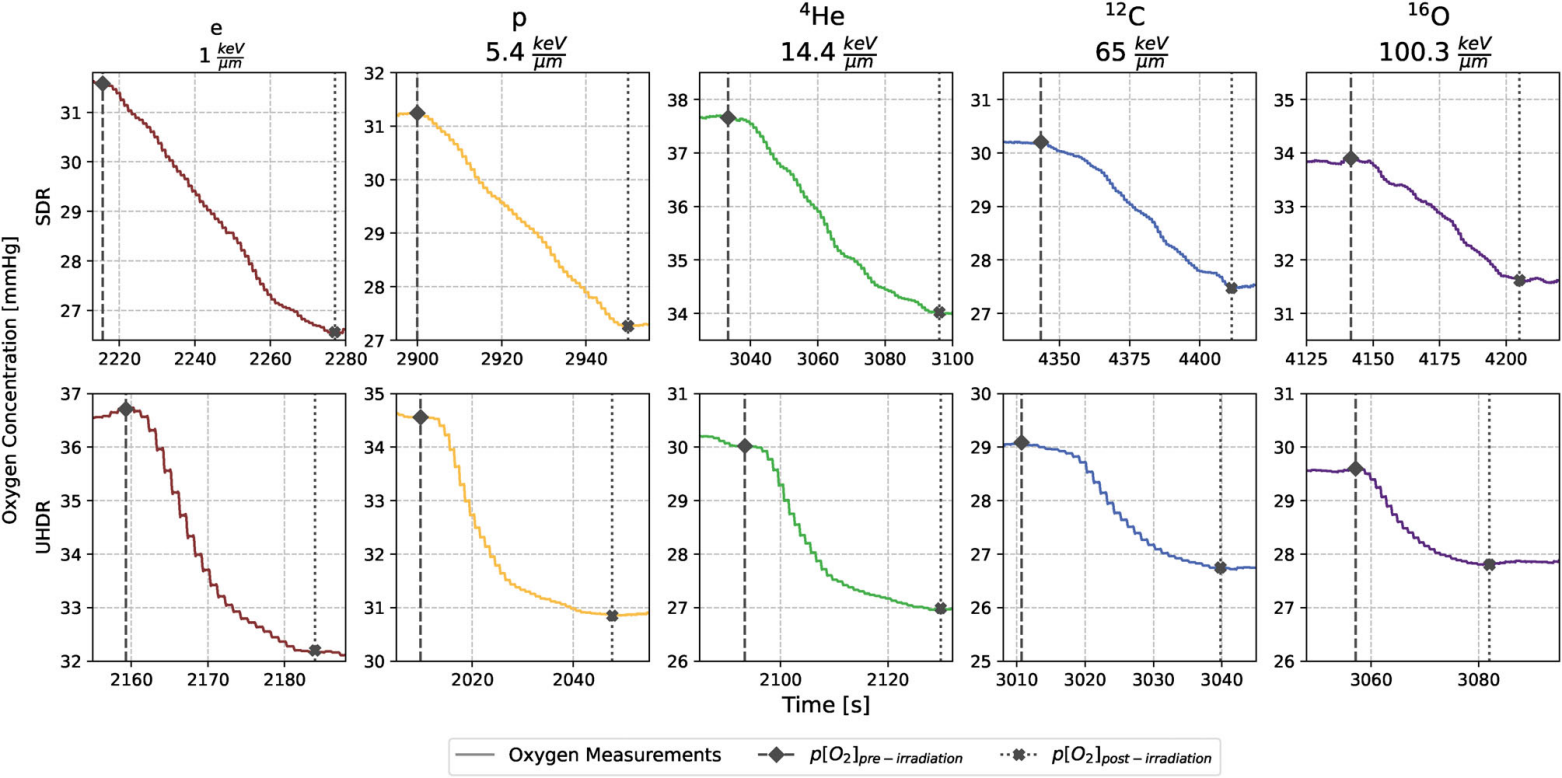
Examples for each particle and dose rate combination of the oxygen concentration over time during a single irradiation. The LETd values are given in the subtitles. p[O_2_]_pre‐irradiation_ is the oxygen concentration marked with a black diamond and a dashed line before the radiation has started, while p[O_2_]_post‐irradiation_ symbolized with a cross mark and a dotted line hints at the start concentration equilibrium after the dose application. The y‐axis always shows a range of 5 mmHg for better comparability, while the time‐axis is chosen to display the whole consumption process. Due to the 20 s response time of the OxyLite, the drop in oxygen concentration after UHDR irradiation is not instantaneous, but rather slowly decreases as the sensor equilibrates with the depleted medium.

At least three independent replicates were evaluated with the recordings from the OxyLite Software LabChart8 (v8.1.19). To assess the oxygen consumption rate *g* [mmHg/Gy], the stabilized oxygen concentration after irradiation $p{\left[O_{2}\right]}_{post-irradiation}$ [mmHg] was subtracted from the concentration before irradiation $p{\left[O_{2}\right]}_{pre-irradiation}$ [mmHg] (Figure 3). This value was divided by the actual applied doses *D* [Gy] given from dosimetric measurements:

(1) $$g=\frac{p{\left[O_{2}\right]}_{pre-irradiation}-p{\left[O_{2}\right]}_{post-irradiation}}{D}$$

The start time of the irradiation was recorded in order to identify the beginning of oxygen consumption in the LabChart files, which corresponds to the point labelled $p{\left[O_{2}\right]}_{pre-irradiation}$ in Figure 3 and Equation (1). Subsequently, the first 20‐second interval where the deviation from the mean oxygen concentration was less than or equal to ± 0.5 mmHg was identified. The start of this interval, which represents a period of stabilization, is labelled $p{\left[O_{2}\right]}_{post-irradiation}$ in Figure 3 and Equation (1). The different *g*‐values from the three replicates were pooled and fitted with a Michaelis‐Menten styled function to correlate the initial $p{\left[O_{2}\right]}_{pre-irradiation}$ with the *g*‐value^14^, ^15^, ^31^:

(2) $$g\left(p{\left[O_{2}\right]}_{pre-irradiation}\right)=\frac{g_{max}\cdot p{\left[O_{2}\right]}_{pre-irradiation}}{k+p{\left[O_{2}\right]}_{pre-irradiation}}$$

Here, *g_max_* and *k* correspond to the plateau *g*‐value, which is reached for high initial oxygen values and to the concentration where half of the *g_max_* is reached, respectively. Thus, *k* also indicates the steepness of the slope. In this context, the fit function describes the variation in oxygen concentration per unit dose. This may be conceptualized as a G‐value, which is defined as the number of molecules consumed per 100 eV of energy deposited, and is employed to describe the rate of reaction.^15^ The fitting was performed with a least squares fit from scipy package optimize.curve_fit (Scipy Version 1.11.1).

## 3 RESULTS

### 3.1 Influence of initial oxygen levels on oxygen consumption rate

**FIGURE 4:**
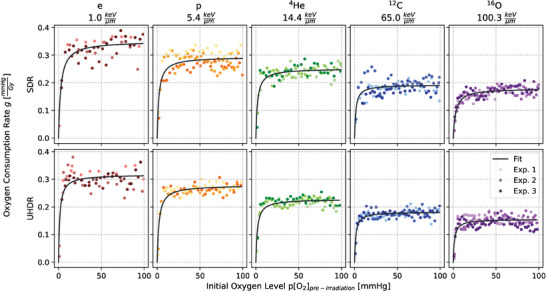
Oxygen consumption rate g in BSA 5% against the oxygen concentration prior to the application of 15 Gy for all the various particle and dose rate combinations. Each shade within a given color chosen for one particle represents an independent replicate and thus experiment (Exp.).

In Figure 4, the measured *g*‐values calculated with Equation (1) are presented in relation to the initial oxygen levels with the fitted function (2). This fit function describes the saturating behavior of the depletion rate *g* for high initial oxygen concentrations and the corresponding fit values can be found in Table 3. While for the slope parameter *k* no trend is observable (Figure S2), *g_max_* shows dose rate and LETd dependencies.

**TABLE 3:** Fit values of the Michaelis‐Menten function for all conditions.

Particle	Dose rate mode	*g_max_* (mmHg/Gy)	*k* (mmHg)
e	SDR	0.351 ± 0.007	2.6 ± 0.6
	UHDR	0.317 ± 0.007	1.5 ± 0.4
p	SDR	0.294 ± 0.005	2.0 ± 0.4
	UHDR	0.279 ± 0.004	2.1 ± 0.3
^4^He	SDR	0.252 ± 0.004	1.9 ± 0.3
	UHDR	0.2282 ± 0.0027	1.74 ± 0.25
^12^C	SDR	0.193 ± 0.004	1.4 ± 0.4
	UHDR	0.1822 ± 0.0019	1.39 ± 0.20
^16^O	SDR	0.1796 ± 0.0022	2.79 ± 0.29
	UHDR	0.1556 ± 0.0022	1.46 ± 0.27

The values are accompanied by their respective statistical uncertainty of the fit represented by the standard deviation.

### 3.2 LETd dependency of oxygen consumption

**FIGURE 5:**
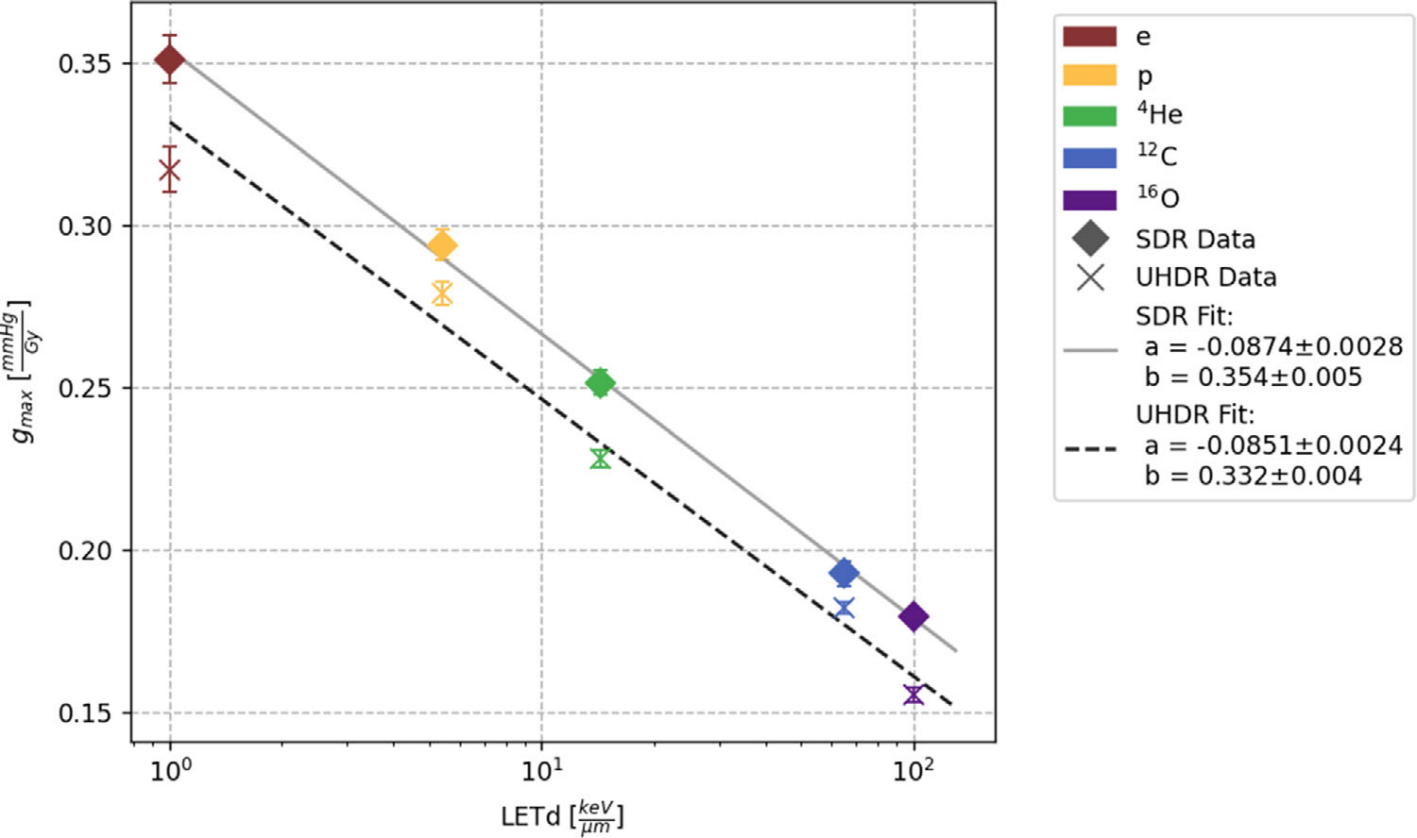
Fitted g_max_ values and their standard deviations for every particle type are plotted against their LETd values. The SDR values are marked with a diamond and fitted by the solid line while the UHDR are represented by a cross and fitted by the dashed line. The fit parameters for equation (III) are given with their statistical uncertainties.

A decrease of *g_max_* with increasing LETd of the particle species can be seen for both UHDR and SDR irradiations (Figure 5). Specifically, electrons exhibit the highest consumption rate with values above 0.3 mmHg/Gy, while oxygen ions with a LETd of 100.3 keV/µm demonstrate the lowest consumption rates below 0.2 mmHg/Gy. A linear function with the logarithm of the LETd:

(3) $$g_{max}=a*log_{10}\left(LETd\right)+b$$

was fitted to the UHDR and SDR data, respectively, and showed a good agreement with the data. The consumption rates of SDR and UHDR separately show a linearly descending trend when the LETd is graphed in logarithmic scale seen in Figure 5. The R‐square values were for both SDR and UHDR fit above 0.98.

### 3.3 Difference in oxygen consumption from SDR versus UHDR consistent with increasing LETd

**FIGURE 6:**
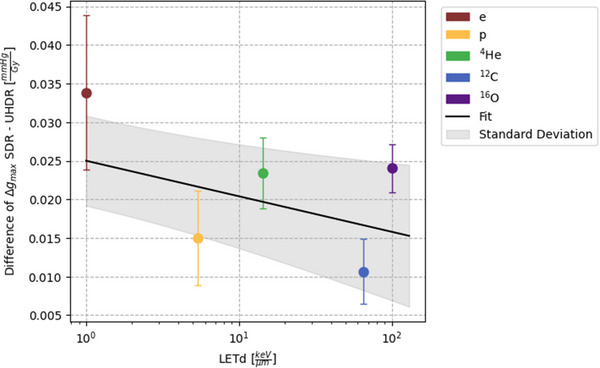
Difference of g_max_ values of SDR and UHDR irradiation for the individual particle beams against their LETd. The values are given with their corresponding standard deviations. Additionally, the difference between the fits for the SDR and UHDR g_max_ values in dependence with LET, as shown in Figure 5, is plotted with its standard deviation represented by the shaded grey area.

For SDR irradiations, the *g_max_* value for each particle is consistently greater compared to the corresponding UHDR value. Since the slopes in Figure 5 for SDR and UHDR are slightly different, we evaluated the evolution of the difference between the *g_max_* values for SDR and UHDR ($\Delta g_{max})$ and the deviation between the SDR and UHDR fits for the *g_max_* and LET correlation (Figure 6). The difference in $\Delta g_{max}$ is slowly decreasing with increasing LETd.

## 4 DISCUSSION

For the first time, we were able to include not only electrons and protons, but also helium, as well as the heavy ions carbon and oxygen for UHDR irradiations to enable a comprehensive study of oxygen consumption for SDR and UHDR over a large span of LETd values in BSA. This direct measurement method revealed that the maximum amount of oxygen consumption given by *g_max_* in BSA decreases with increasing LETd. Furthermore, the amount of oxygen consumption was found to be smaller for UHDR in comparison to SDR, throughout the LETd range.

The differences in oxygen consumption associated with LETd may be due to altered radiochemical reactions occurring at the molecular level. This nanoscale interpretation presents a significant challenge, as these reactions are inherently difficult to measure directly due to their short‐lived nature, typically occurring on timescales of less than 1 µs. Computational tools such as TRAX‐CHEM,^32^ IONLYS‐IRT,^33^ or Geant4‐DNA^34^ have demonstrated the ability to model these intricate reaction patterns, although limited to pure water environments and single track simulations.^32^, ^33^, ^34^

Our results agree qualitatively with previously conducted in silico studies performed with MC simulations in water, which also found that higher LET result in lower oxygen consumption rates. More specifically, Boscolo et al. demonstrated in silico that the overall yield of chemical species generated during high LET irradiation is reduced compared to low LET conditions.^19^

While inter‐track interactions can be neglected for the investigated LET during SDR irradiation,^35^ the elevated LET values may correspond to an increased density of chemical reactions within the track. This increase in spatiotemporal proximity of radicals for high LET and consequent acceleration of intra‐track reaction kinetics enhances the likelihood of radical recombination and self‐interaction between the primary chemical species. About 1 ps after the initial irradiation, the primary species are mainly HO•, e^‐^_aq_, H•, and H_3_O+.^19^, ^36^ As the LET increases, the spatial distance between these radiation products decreases within the track, facilitating interaction between the radicals, like the self‐interactions H• + H• and HO• + HO•, which lead to more molecular species H_2_ and H_2_O_2_, respectively.^19^ Furthermore, the radical recombination, HO• + e^‐^_aq_, becomes the dominant reaction over HO• + O₂ due to this increased density of the two radicals. The overall G‐values of HO‐, e^‐^_aq_,H_3_O+, and peroxide radicals decrease with increasing LET due to radical interactions.^32^, ^33^, ^36^ These examples highlight how self‐interaction and radical‐radical recombination decrease the interaction rate with molecular oxygen, leading to reduced oxygen consumption during high LET irradiation.^19^ The effect of oxygen consumption may also be marginally minimized by the simultaneous generation of oxygen within high LET particle tracks. The “Oxygen in Track Hypothesis” describes the creation of an oxygen‐enriched microenvironment around the high‐dose track core of ions.^33^ According to Meesungnoen et al., the oxygen produced during irradiation primarily affects the early stages of chemical reactions (around 1 µs post‐irradiation).^33^ In contrast, Boscolo et al. report that the yield of oxygen consumption is 500 times higher than the production of oxygen at the same time point and LET value.^19^ Considering the difference in the simulation approaches, the difference in production and consumption yield would suggest that the oxygen produced has a minimal effect on the total oxygen concentration seconds after the irradiation. The measurements conducted in this study reflect the overall changes in oxygen concentration, suggesting that the reduced oxygen consumption at high LET may be due to the inherently lower reaction rate with molecular oxygen, primarily due to radical‐radical and self‐interaction, and to a lesser extent the oxygen production within the tracks.^19^, ^33^

The data obtained in this study, with its wide range of LETd values, might act as a valuable benchmarking resource for future developments in radio‐chemical MC simulations, which could enable more accurate predictions of the oxygen‐dependent reactions following ionizing radiation.

The rate of consumption is observed to be lower for UHDR irradiation compared to SDR irradiation. For lower LET irradiation, this behavior was already observed in several studies using water,^15^ different BSA concentrations,^14^, ^31^ and solutions that mimicked the intracellular chemical milieu with higher fidelity (namely “CELL”).^18^

This difference between SDR and UHDR may be attributed to the accelerated temporal application of particles, which could promote inter‐track interactions. Inter‐track radical‐radical reactions and self‐scavenging of radicals would lead to a reduce the likelihood of interactions with oxygen molecules present in the sample.^19^ Nevertheless, some MC studies have indicated that such inter‐track interactions may not be the underlying mechanism responsible for the observed decrease in oxygen consumption rates,^35^, ^37^, ^38^ while other studies have suggested that, under specific conditions, such as the administration of large doses and the utilization of low‐LET, inter‐track interactions at UHDR may potentially contribute to the enhancement of radical interactions.^20^, ^39^, ^40^ Given the ongoing debate surrounding the fundamental parameters of these analyses, including the diffusion constant for radicals within cells, further research is required to elucidate the underlying mechanism behind the UHDR‐mediated reduction in oxygen consumption.

In this work, we demonstrated that with increasing LETd, the discrepancy between UHDR and SDR consumption rate slightly declined. As LETd and UHDR increased gradually, the radical‐scavenging effect may have reached a saturation point, resulting in a reduction in the difference between SDR and UHDR. This hypothesis may be supported by the slightly smaller decreasing slope of the UHDR consumption rate fit. To investigate this further, measurements with an even higher LETd would be required.

Hence, both high LET and UHDR individually may result in closer spatiotemporal proximity of radicals and lead to radical‐scavenging and thus a reduced interaction rate with molecular oxygen in the sample.^19^, ^33^, ^36^

This radical scavenging could be beneficial for cell survival by reducing oxidative damage to key cellular biomolecules.^16^, ^41^ Normal cells possess an advantage over tumor cells due to their lower prooxidant burden and higher reserves of antioxidant enzymes and pathways.^42^ This enables normal cells to rapidly and efficiently eliminate remaining radicals, even before the initiation of further harmful Fenton‐type reactions or peroxidation chains, providing superior protection against further damage in contrast to tumor cells, which lack these robust cellular defense mechanisms. Consequently, the differential antioxidant capacities of normal and tumor cells may contribute to their varied responses to UHDR, where the total amount of radicals might be reduced.^13^

Our analysis was based on the plateau parameter *g_max_* obtained by fitting the *g*‐values to a Michaelis‐Menten curve. The second fit parameter *k* correlates with the oxygen concentration that yields half the maximum consumption rate and thus with the slope of the initial increase. The smaller this value, the faster the plateau value *g_max_* is reached. No clear LETd trend or dose rate effect can be seen in the data (Figure S2). However, due to the inherent resolution limitations of the OxyLite, measurements at concentrations below 7 mmHg are more prone to error with a resolution of ± 0.7 mmHg. This resolution limit could theoretically be overcome by using phosphorescence measurement methods due to their longer lifetimes and correspondingly higher accuracy in low oxygen environments. Despite this, given the additional small number of data points in the low oxygen concentration region, the fit of the *k* parameter is associated with larger standard deviations (Table 3), which hinders sensible interpretation of the data. Furthermore, the response time of the OxyLite system is limited to 20 s, a characteristic common to detectors embedded in membranes, such as the TROXsP5.^43^ However, the longer response time was considered sufficient for our purposes, as it enables the evaluation of the total oxygen changes caused by the applied beams (Figure 3).

While several studies of oxygen consumption for electrons and protons have been conducted,^14^, ^18^, ^31^, ^44^ only one study in literature measured the consumption rate in deionized water after carbon ion irradiation. However, the LET reached in this experiment was relatively low at 19.47 keV/µm and so was the dose rate with a maximum of 1.8 Gy/s.^45^ Since deionized water is not recommended as surrogate for cellular milieu,^17^, ^41^ in this study, BSA was used as a surrogate for the extracellular environment.^18^ The simplicity, stability, and reproducibility of this solution allowed us to discern the slight differences in oxygen consumption between each radiation quality. However, in comparison to actual cells, no thiols, lipids, or scavengers are present in the BSA solution. Slyker et al. demonstrated that in the “CELL” solution, the consumption rates were higher than in BSA but still showed the same dose rate trend. While the applicability of BSA is surely limited, it gives a first hint on how the consumption rate changes with dose rate and LET in a medium with proteins and a high percentage of water.^18^ The tendency of a higher consumption for SDR compared to UHDR was already found in other experiments using BSA solutions.^14^, ^31^, ^44^ Our experiment is qualitatively consistent with their findings and provides an extrapolation to higher LETd. Differences in the quantitative values may be caused by deviations in the irradiation setups.

A recent study by Khatlib et al. directly measured intracellular oxygen consumption.^46^ Notably, this study observed the disappearance of the disparity in oxygen consumption rates between SDR and UHDR irradiations against the expectancy that organic substances would enhance the oxygen consumption. This phenomenon could be explained by cellular antioxidant mechanisms, such as catalase and superoxide dismutase reactions, which convert radicals back to oxygen and potentially compensate for oxygen consumption.^46^ Additionally, several reducing agents influence the consumption rate in different ways. For instance, glutathione enhances it, while ascorbate decreases it, as demonstrated by Koch et al.^17^ To decipher the complex radiochemistry behind the radiochemical oxygen consumption and whether the LETd effect on oxygen consumption also disappears intracellularly or even in actual tissue needs to be examined in further studies.

If oxygen consumption and the following transient hypoxia was one of the underlying mechanisms of UHDR sparing, our findings would suggest that sparing would decrease for higher LETd. While separate studies of in vitro UHDR‐sparing effects after ion beam irradiations exist, namely for helium^28^ and carbon^25^ ions, comprehensive data sets ranging over a substantial LET‐region are still lacking. In combination with the results of our investigations, such in vitro datasets could allow a test of biophysical models related to radiochemical oxygen change.

Regarding the in vivo FLASH effect, a number of low‐LET electron and proton studies are available for consideration.^47^, ^48^, ^49^ Furthermore, Tinganelli et al. presented evidence of the benefits of lower LET (14.5–15.5 keV/µm)^50^ and recently also for high LET (65–85 keV/µm)^51^ carbon ion irradiation at UHDR in vivo. Consequently, high LET data is also a crucial area of investigation in vivo and could lead to profound implications for FLASH treatments with heavy ions. In heavy ion radiotherapy, the target is usually exposed to elevated LET, while the normal tissue is predominantly receiving low LET radiation. Given the LET dependence of oxygen consumption observed in this study, in tumor cells subjected to high LET irradiation less oxygen would be consumed. This would be advantageous, as it reduces the likelihood of sparing tumor cells. Furthermore, biological efficacy of high LET irradiation in general is known to be less influenced by the oxygen environment, since the densely ionizing tracks induce a higher proportion of direct damage and rely less on the indirect processes.^52^, ^53^ This could even further decrease the biological effect of the theoretically expected marginal oxygen consumption induced sparing. For the normal tissue exposed to low LET irradiation, increased oxygen consumption would refer to a beneficial larger sparing

Although the oxygen consumption rate of UHDR is slightly lower than that of SDR irradiation, this rate only includes oxygen consumption, not the associated reoxygenation processes, which is substantially changed after irradiation of UHDR and SDR due to their time scale differences. The advantage of UHDR is that oxygen consumption happens so quickly that cells remain in a state of reduced oxygen until reoxygenation can compensate for the oxygen loss. Conversely, during SDR irradiation, oxygen consumption is directly compensated by reoxygenation due to the longer irradiation duration.^11^, ^12^, ^54^

Consequently, this study indicates that heavy ion radiotherapy at UHDR may further expand the therapeutic window by ensuring higher consumption rates and thus sparing in normal tissue while providing increased radiobiological effectiveness in the target region.^38^, ^50^, ^55^ One may even consider enhancing this effect by employing strategies to maximize the LET in the target region, since higher LET corresponds to lower oxygen consumption.^56^

## 5 CONCLUSION

This study presents the first direct measurements of the oxygen consumption rates in BSA 5% samples with different particles over a wide range of LETd up to 100.3 keV/µm. Using the HIT synchrotron, we were able to perform UHDR irradiations with protons, helium, carbon ions and, for the first time, oxygen ions. The results show a systematic decrease of the oxygen consumption rate with increasing LETd. In addition, the UHDR consumption rate remains consistently lower than the corresponding SDR consumption rate.

Through its comprehensiveness, our dataset could be a valuable benchmark resource for radiochemical MC simulations and oxygen dynamics models. Comparison of the simulation results with our empirical findings could enhance the understanding of the combined effects of high‐dose rates and increased LETd in relation to oxygen‐dependent reactions after irradiation.

In combination with additional data on the trend of sparing effects over the LETd, our results may aid the appraisal of key candidates for the underlying mechanism of UHDR sparing and eventually the FLASH effect.

If the sparing phenomenon persists in higher‐order biological samples and correlates with the oxygen consumption trends observed in this study, it suggests potential synergies between heavy ions and UHDR. In this scenario, high consumption rates may confer protection in normal tissues and organs at risk, while the high LET in the target would ensure reduced consumption and thus may avoid potential sparing of the tumor.

## CONFLICT OF INTEREST STATEMENT

Jürgen Debus reports grants from CRI—The Clinical Research Institute GmbH grants from View Ray Inc., grants from Accuray International Sarl, grants from Accuray Incorporated, grants from RaySearch Laboratories AB, grants from Vision RT limited, grants from Merck Serono GmbH, grants from Astellas Pharma GmbH, grants from AstraZeneca GmbH, grants from Siemens Healthcare GmbH, grants from Merck KGaA, grants from Solution Akademie GmbH, grants from Ergomed PLC Surrey Research Park, grants from Siemens Healthcare GmbH, grants from Quintiles GmbH, grants from Pharmaceutical Research Associates GmbH, grants from Boehringer Ingelheim Pharma GmbH Co, grants from PTW‐Freiburg Dr. Pychlau GmbH, grants from Nanobiotix AA, outside the submitted work. Amir Abdollahi and report grants and other from Merck and EMD, grants and other from Fibrogen, other from BMS, other from BioMedX, other from Roche, outside the submitted work.

## Supporting information



Supporting Information

Supporting Information

Supporting Information

## Data Availability

Research data is stored in an institutional repository and will be shared upon request to the corresponding author.
