# Supplementary figures and images for: Oxygen consumption measurements at ultra‐high dose rate over a wide LET range
Source: Med Phys. 2024 Nov 6;52(2):1323–34. doi: 10.1002/mp.17496 (PMC11788059; doi:10.1002/mp.17496)

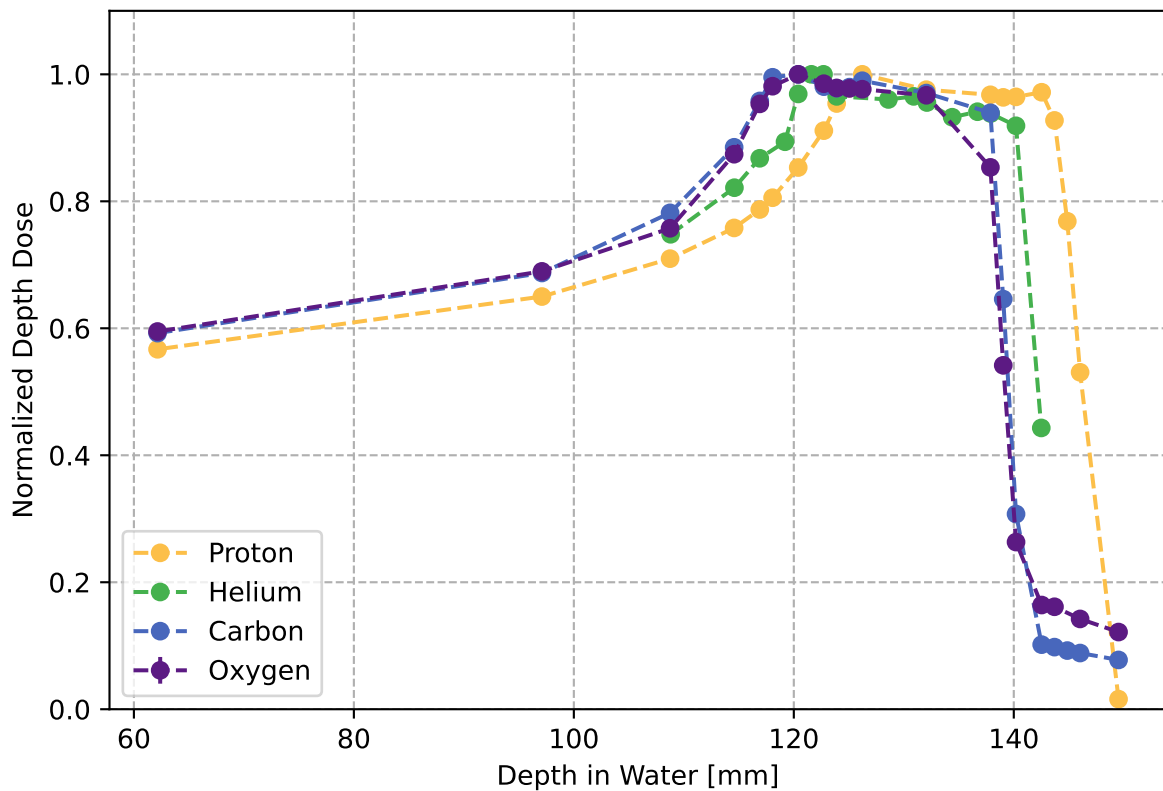

Supplement: Supplementary file 1 — Supporting Information [file MP-52-1323-s001.pdf]

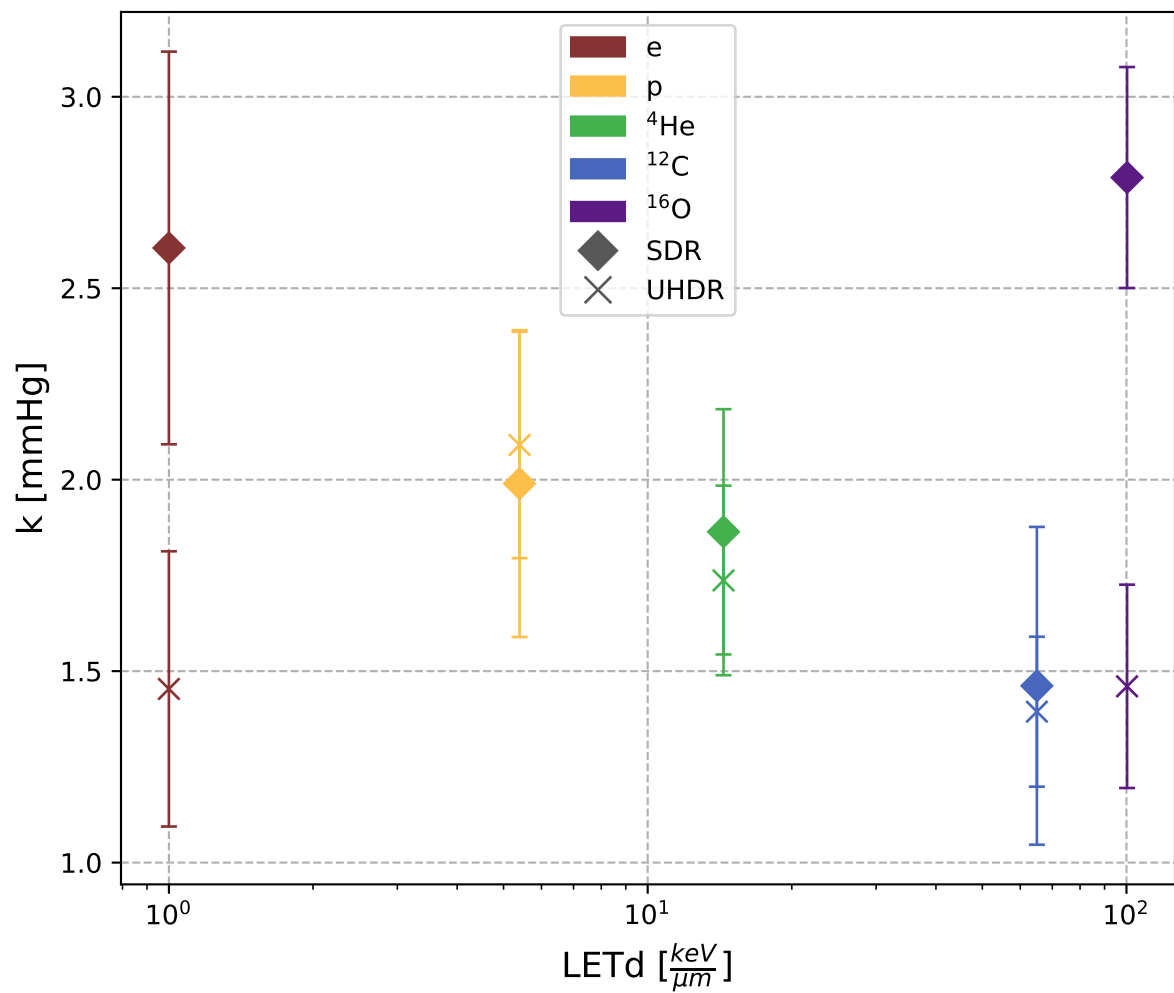

Supplement: Supplementary file 2 — Supporting Information [file MP-52-1323-s002.pdf]
