# Supplementary material for: Oxygen consumption measurements at ultra‐high dose rate over a wide LET range
Source: Med Phys. 2024 Nov 6;52(2):1323–34. doi: 10.1002/mp.17496 (PMC11788059; doi:10.1002/mp.17496)
Supplement: Supplementary file 3 — Supporting Information [file MP-52-1323-s003.pdf]

Supplementary

Figure Captions

Figure S 1: Depth dose curves for the 2DRM obtained with the beam parameters mentioned in Tabel 2 for the UHDR irradiations. The normalized depth dose is plotted against the depth in water. Due to the different energies of the primary particles the curves are shifted in depth, which is accounted for in the experiment by using different thicknesses of PMMA.

Figure S 2: Fitted k values and their standard deviations for every particle type against their LETd values. The SDR values are marked with a diamond while the UHDR are represented by a cross.
